# The Effects of Thymoquinone Loaded in Alginate Scaffold on Bone Regeneration in Rabbit Mandible Defect

**DOI:** 10.31661/gmj.v12i.3141

**Published:** 2023-12-17

**Authors:** Ehsan Aliabadi, Mohammad Mehdi Farahmand, Tahereh Talaei-Khozani, Sheila Shahsavari-pour

**Affiliations:** ^1^ Department of Oral and Maxillofacial Surgery, School of Dentistry, Shiraz University of Medical Science, Shiraz, Iran; ^2^ Histomorphometry and Stereology Research Center, Department of Anatomy, School of Medicine, Shiraz University of Medical Science, Shiraz, Iran

**Keywords:** Bone Regeneration, Thymoquinone, Alginate

## Abstract

Background: Thymoquinone (TQ) has some beneficial roles in bone repair. Local administration of the drugs by loading them into a scaffold leads to releasing higher concentrations of a drug in the appropriate position. This study was conducted to evaluate the effects of the local administration of TQ loaded in an alginate scaffold on bone regeneration in rabbit mandibular defect.Materials and Methods: In this experimental study, male rabbits with mandible defect were divided into 3 groups that received either alginate containing 50µM TQ, alginate or remained untreated. Then each group was subdivided into 2 groups that followed for 4 and 8 weeks (n=5). The radiological opacity, histopathology, and histomorphometrical analysis were done and data were analyzed by ANOVA and Tukey. Results: Radiological examination indicated that the presence of TQ had no significant effect on the turbidity of the images (P=0.595). TQ treatment significantly increased the bone area after 4 (P=0.011, P=0.0021, respectively) and 8 weeks (8w P=0.019, P0.001, respectively) compared to alginate-treated and control defects. TQ also elevated the number of osteocytes (4wP0.001, P=0.001 and 8w P0.001, P0.001, respectively) and osteoblasts (P0.001, P0.001, respectively) compared to the group receiving alginate and control. At 8 week, although the average number of osteoblasts in the defects treated with TQ was significantly higher than the control group (P=0.01), it was similar to the group that received alginate.Conclusion: It seems that alginate containing TQ has positive effects on bone healing. TQ can be suggested as a good additive to improve bone healing and accelerate bone repair.

## Introduction

Under appropriate physiological conditions, spontaneous repair happens in most of the bone defects due to the high capacity of regeneration. However, because of the reduced blood supply at the fracture site and calcium and phosphorus deficiency to reinforce the newly formed bones, the bone healing process is time-consuming. In addition, large defects, greater than critical bone defect size, may fail to regenerate spontaneously due to weak biomechanics, abnormal conditions in the wound site, poor surgical technique, and factors such as changes in metabolism, hormones, and nutrition [1]. Bone grafting or biomaterial substitutes are commonly used in the reconstruction and filling of large bone defects. Although autografts are the current gold standard treatment for bone defect reconstruction [2][3], they still have disadvantages such as donor supplies [4], pain at the donor site, or bleeding [5].

Allograft transplantation has some disadvantages such as a higher chance for immune rejection, infectious disease transmission, and adverse effects on the mechanical and biological properties of the graft [6][7]. To overcome these limitations, using biomimicry materials has been suggested in the last decades [8][9]. An ideal scaffold for bone graft should be biocompatible, bioabsorbable, osteoconductive, biomimicry, and user-friendly. Before testing on humans, an ideal bone substitute should be tested in vitro and in vivo to ensure that it works effectively and safely. Therefore, developing a suitable animal model is an essential step in evaluating the effectiveness of bone substitute biomaterials [10]. Thymoquinone (TQ) is known as a volatile bioactive component in black seed oil (Nigella Sativa). TQ acts as an antioxidant and mediates some roles in the activation of osteoclasts, which are especially involved in bone resorption [11]. Due to its antioxidant properties [12], TQ plays an important role in accelerating bone formation. The systemic use of TQ accelerates new bone formation in the rapid mid-palatal expansion method, so it was thought that the systemic use of TQ may have a potential therapeutic effect [13].

A study by Alkhatib et al, investigated the effects of TQ as a supplement to promote the healing and regeneration of periodontal tissue [14]. Along with clinical trials, laboratory studies show the beneficial effects of TQ during periodontal treatment [11]. Although in low doses, TQ reduces hepatotoxicity [15], the toxic effects of high doses were shown, in animal studies previously. Oral administration of 250-794 mg/kg in rats and 300-2400 mg/kg in mice was considered as LD50. Besides, the bioavailability of the TQ is about 58% in rabbits and showed rapid expulsion following oral treatment [16].

TQ has limited solubility and bioavailability, and loading into a scaffold can modulate the release, increase the local bioavailability, and decrease the high dose toxicity [17]. Alginate, an anionic polysaccharide polymer, is becoming more applicable in bone tissue engineering due to its biocompatibility and gelling properties [18].

Due to its availability, cheapness, and biocompatibility, alginate is considered a popular scaffold that delays or regulates drug release [19]. Besides, alginate-based composites induce cell proliferation, as well as an increase in the alkaline phosphatase activity in bone cells, excellent mineralization, and bone differentiation. Therefore, the use of alginate-based composite biomaterials for bone tissue reconstruction will lead to promising results [20].

The rabbit is one of the most commonly used animal models, ranking first among all animals used for musculoskeletal system research [10]. However, considering the evaluation of several alternative biomaterials, the small size of rabbits is a major drawback for the study of orthopedic implants. In addition, compared to other species, such as primates or some rodents, rabbits undergo faster skeletal changes and bone turnover [21]. Rabbits are readily available and easy to keep. These characteristics make rabbits the first choice for in vivo testing of new bone substitute biomaterials [22].

A previous in vitro study showed that TQ loaded on alginate-hydroxyapatite composite scaffold can increase the activity of MG63 bone cells and increase the expression of specific bone markers such as osteopontin, osteocalcin, and collagen. In addition, it also accelerates the process of mineralization [23]. However, the use of TQ in the in vivo environment as a substitute for bone tissue was not evaluated. Therefore, the present study was conducted to evaluate the effects of the TQ-loaded alginate on bone regeneration in rabbit mandibular defects.

## Materials and Methods

In this experimental study, 30 New Zealand white male rabbits at the age of three months and weighing about 2 kg were purchased from the animal shelter of Shiraz University of Medical Sciences. The animals were treated according to the ethics University committee’s guidelines IR.SUMS.AEC.1401.040. The rabbits were housed individually in standard cages (6 x 45 x 45 cm) under controlled temperature (22-24°C) and 12.12 hours of light-dark cycle and ad libitum to induce adaptation.

Study Design

The animal models with mandibular defect were divided into 3 groups; those treated with TQ-loaded alginate, those that received alginate and those that remained untreated. All groups were subdivided into a 4-week and an 8-week follow-up group (n=5). The defect of the control group was just covered with the superimposed tissues including periosteum, muscles, and skin without any treatment.

Surgical Procedure

The rabbits were injected intramuscularly with xylazine 2% (Alphasan, WOERDEN-HOLLAND), at a dose of 3.5 mg/kg and ketamine 10% (BREMER PHARMA GMBH 34414, Warburg, Germany) at a dose of 50 mg/kg, at a ratio of 3 to 1. After induction of anesthesia, the hair of the lower jaw was shaved and disinfected using 10% povidone-iodine. Lidocaine 2% HCl (ZEYCO, 401 G1 9020, Mexico) was simultaneously used for local anesthesia and pain reduction during surgery. To prevent possible damage and appearance of mandibular bone, mandibular skin was cut and the main muscle was contracted. A cube defect with a width of 15 mm, a height of 10 mm, and a depth of 4 mm was created unilaterally in the lower jaw using dental burs and simultaneous irrigation with normal saline. During the surgery, an electric blanket was used to prevent the death of the animal.

Preparation of the Scaffolds

To prepare the scaffold, a 1% sodium alginate was prepared in normal saline. According to the previous in vitro study [23], 50 µM TQ was used to construct the scaffold. Electrogelation of the alginate and TQ-loaded alginate was done by adding 1% calcium chloride. The defect site was filled by hydrogels, and the incision was sutured. The sutured area was disinfected with oxytetracycline (OTC) as a disinfectant after the operation. The operated rabbits woke 1 hour after the operation and were transferred to isolated cages with free access to food and water for 4 and 8 weeks. To control analgesia, 0.22 mg/kg of morphine was prescribed. Morphine causes long-term postoperative analgesia in the epidural space [24]. Also, in the first 3 days after the operation, intramuscular injection of penicillin/streptomycin was done daily. At the end of the study, the animals were killed using an overdose of CO2 gas in a euthanasia chamber. Their lower jaw bone was removed and the results of the study were analyzed.

Histological and Radiological Evaluations

X-ray photography was done using an X-ray machine (Planmeca Intra, Finland). The intensity of brightness in radiology images was evaluated using ImageJ software (developed at the National Institutes of Health and the Laboratory for Optical and Computational Instrumentation) (http://imagej.nih. gov/ij/index.html). In this method, the brightness is evaluated at the range of 0 and 255 at any point. The average of this number in all selected points shows the average brightness. Next, the bones were fixed in 10% formalin buffer and decalcified for 3 days in a solution containing 8% HCl and 8% formic acid.

After routine tissue processing, the samples were cut into 5 µm slices. Three sections from the beginning, middle, and end of tissues were selected. All slides were stained with hematoxylin and eosin. Areas all slides were photographed using a systematic random sampling method with a magnification of 10. Finally, the total newly formed bone and connective tissue area, the number of osteoblasts and osteoclasts were measured by using ImageJ software.

Statistical Analyses

First, the normality of the data was checked by the Kolmogorov Smirnov test and they followed the normal distribution. Then, the normalized data was analyzed by one-way analysis of variance ANOVA. The follow-up test used in the study was Tukey’s test. To analyze the data, SPSS 20 (IBM, USA) was used. The graphs were depicted by Prism. The significance level in the study was 0.05.

## Results

**Figure-1:**
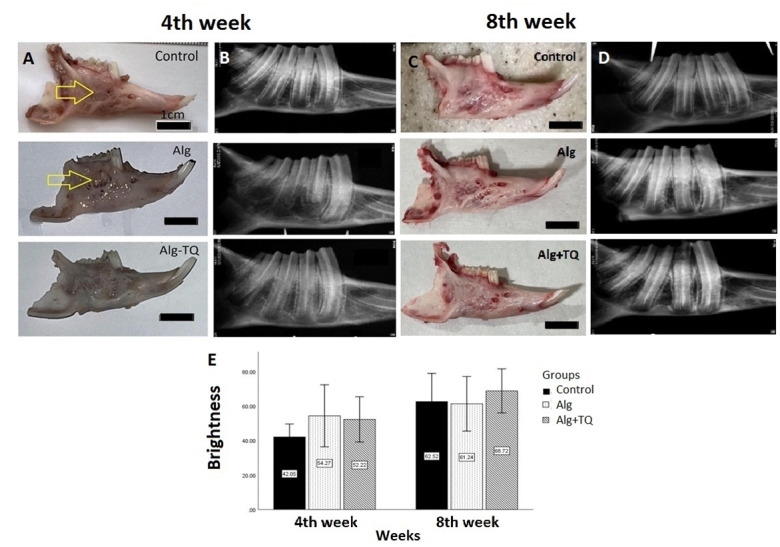
Figure 1. comparison of the macroscopic and radiological evaluations of the different groups. Arrows indicates the repaired defect

**Figure-2:**
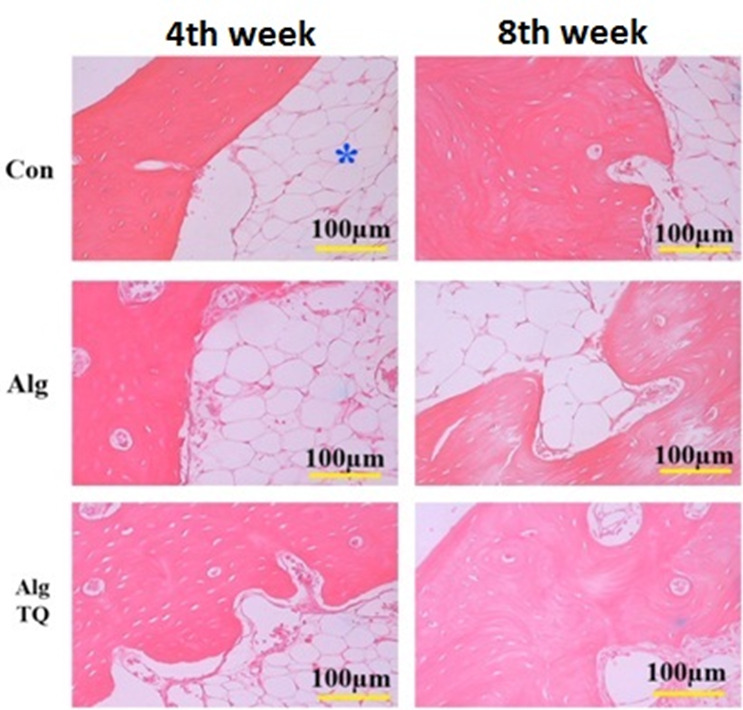
Figure 2. Comparison of histological evaluation of a representative sample from each group. In all defects, the newly formed bone, connective tissue and adipose tissue (*) can be observed. Alg: alginate; Alg+TQ: alginate and thymoquinone.

**Figure-3:**
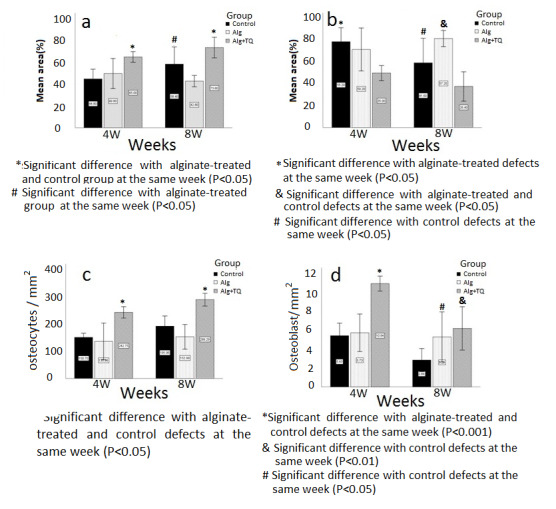
Figure 3. Comparison of the average bone area (A), the average area occupied by connective tissue (B), the average of the number of osteocytes (C) and osteoblasts (D) per square millimeter of the tissue surface in 4th and 8th week. Alg: alginate; Alg+TQ: alginate and thymoquinone

Macroscopic evaluation showed that the gross appearance of bone regeneration and recovery in the area receiving the TQ-loaded scaffolds was better than the alginate-treated and control groups. Also, alginate had no beneficial impact on the gross appearance of bone defects compared to control defects (Figure-1).

The radiological examination showed that the presence of TQ had no significant effect on the turbidity of the images (P=0.595). Figure-1E compares the mean radiographic luminance of different groups at weeks 4 and 8. According to the graph, after 4 weeks, the highest average radiographic brightness was observed in the group receiving alginate scaffold alone with an average of 54.27±14.49 and the lowest average radiographic brightness was observed in the control group with an average of 42.05±6.04. This difference was not statistically significant (P=0.208). After 8 weeks, the highest average radiographic brightness was observed in the group receiving TQ-loaded alginate scaffold with an average of 61.71±10414.29 and the lowest average radiographic brightness was observed in the group receiving alginate scaffold alone with an average of 61.23±12.75. This difference was also not statistically significant (P=0.595). This may indicate that TQ had no significant impact on the mineralization of the newly formed bone (Figure-1).

Histological Results

Figure-2 compares histological evaluation in the different groups. Histopathological evaluation showed that the grafted scaffolds were completely absorbed after 4 and 8 weeks in all groups and were replaced by bone and connective tissues. Connective tissue includes dense connective tissue and fat tissue. No inflammation or invasion of inflammatory cells were observed in the sections of different groups. The presence of blood vessels with different sizes was also seen in the sections (Figure-2).

Morphometric Results

According to the graph in Figure-3A, 4 weeks after the treatment, the statistically highest average percentage of the bone area with respect to the total defect area belonged to the group treated with TQ-loaded alginate scaffold (65%±3.93), and the lowest average is related to the control group ( 8%±7.22)(P=0.005). Also, after 8 weeks, the group treated with TQ-loaded alginate scaffold had a significantly highest bone area (3.60%±7.60), compared to the group receiving alginate (42.8%±4.20, P=0.001) and control (58.4±12.73, P=0.019). In the long term, bone formation in the alginate-treated group was significantly (P=0.017) less than control.

According to Figure-3B, in the fourth week, connective tissue including adipose tissue and fibrous tissue significantly reduced in the defects received TQ-loaded alginate (35±3.93) compared to the control group (55.20%±7.22, P=0.005). In the eighth week, the connective tissue was significantly higher in the group receiving alginate (57.20±4.20) compared to the group treated with TQ-loaded alginate scaffold (26.40±7.60, P=0.001).

As the Fig 3C shows, at both points of time, TQ loading led to a significant increase in the number of differentiated osteocytes (for the 4th week, 242±16.87/mm2; for the 8th week 289.28±18.91/ mm2 ) compared to the group receiving alginate ( for 4 week 135.96±54.22/mm2, P=0.003; for 8 week 152.99±36.62/mm2, P<0.001), and control (for 4 week 150.69±12.35, , P=0.001; for 8 week 191.95±30.18, P=0.001 ). The defects treated with alginate or that remained untreated (control) had similar number of osteocytes.

As Figure-3D shows, at both points of time, the number of osteoblasts was significantly higher in the defects filled with TQ-loaded alginate (for the 4th week 10.94±0.63/mm2, for the 8th week 6.19±1.85/mm2) than control group (for 4 week 0.63 5.43±1/mm2, P=0.01; for 8 week, 2.84±0.98/mm2, P=0.026). Although the average number of osteoblasts in the group receiving alginate scaffold (for the 4th week, 5.73±1.6/mm2) was also significantly higher than the control group (P=0.045) in a long time, it was statistically similar to the group receiving TQ-loaded alginate (P=0.42).

## Discussion

The current study shows TQ accelerates bone formation. Thymoquinone, has antioxidant, anti-inflammatory [25], antitumor, immunomodulatory, bronchodilator, blood pressure lowering, antidiabetic, hepatoprotective, digestive protective, antihistaminic, antimicrobial, and protective effects [15][26][27]. However, recently, much attention has been paid to its beneficial effects on bone formation and recovery through effects on bone cell metabolism and ossification. Systemic TQ administration to a mouse model for rapid maxillary expansion [13] and tibial bone defect [11] showed boosting bone and capillary formation. In vivo application of TQ also reduced bone absorption and [28] and showed anti-apoptotic effects that prevent osteonecrosis [29].In vitro TQ treatment showed an increase in the proliferation and differentiation of MC3T3-E1 osteoblasts cell line and mineralization [23]. An in vitro study shows TQ increases the differentiation of osteoblasts without significantly affecting the physical and mechanical properties of the scaffold [23]. With a wide range of activities and biological applications, quinones have several beneficial effects on bone formation. In this regard, both animal and human studies showed that quinones have beneficial effects on osteoporosis conditions [30][31]. Quinones exert some physiological functions on bone tissue such as inhibiting bone absorption, decreasing the activation of osteoclast, and inducing osteoblast proliferation and differentiation. All of these events lead to bone resorption [32][33]. Our study evaluated the in vivo effects of TQ application in combination with alginate on bone formation. Rahmani Moghadam et al. [23] also confirmed that TQ loading on HA/alginate scaffolds effectively accelerates the in vitro osteoblastic differentiation of mesenchymal stem cells (MSC) that can be used in bone tissue engineering. It was already well known that both hydroxyapatite and alginate have osteogenic effects on MSC differentiation [34]. Alginate has also been used as a suitable drug delivery vehicle. The research has shown that the biological properties of TQ can be modified by incorporation into a suitable drug delivery system. For example, it has been reported that the anti-proliferative or anti-inflammatory activities of TQ are enhanced by encapsulation in polylactide-co-glycolic acid nanoparticles [35]. Also, the antioxidant activity of TQ has been found to be higher when loaded into poly (sodium N-undecylenoyl-valinate) particles than that of free TQ [35]. One of the possible reasons for the above observations is the instability of the TQ molecule in aqueous solutions [36]. Encapsulation of TQ in alginate has been reported to increase its stability [37]. Morphometrical data of the current study showed an increase in the newly formed bone area and the number of osteocytes and osteoblasts by adding TQ to the transplanted scaffolds in the jaw bone defects of rabbits These results show that TQ acts as an osteoconductive agent and induces the host cell migration to the TQ releasing site and therefore has a positive effect on bone healing. In vitro administration of TQ has been reported to accelerate osteoblast proliferation and differentiation and elevate alkaline phosphatase activity and bone extracellular proteins such as collagen and osteonectin [23]. This data is in line of our findings.

The radiological report revealed an increase in the opacity of the defect area in each individual group as time progressed. The opacity can be a sign of mineralization. Therefore, it seems that the mineralization increases with time. However, our data revealed TQ has no significant influence on mineralization. In contrast with our, in vitro data showed TQ increased mineralization of differentiated osteoblasts from MSCs [23]. The presence of hydroxyapatite in the TQ-loaded alginate in that study may be the reason for such controversy.

The results of this study are in accordance with the studies of Mohammed et al. who reported that Nigella sativa promotes bone healing by inducing rapid production of bone trabeculae and mature bone formation [38]. Another recent study showed that TQ accelerates bone formation and reduces the retention period in rapid maxillary expansion [13]. Also, in line with our study, TQ has been shown to increase the amount of newly formed bone, the number of osteoblasts and capillary density in the tibial bone defects of rats [39][40]. Histomorphometry study showed that intraperitoneal injection of TQ also increased the bone volume and decreased the connective tissue volume [41] which is in the line of our data. An animal study conducted on femural fracture repair showed that TQ may facilitate bone repair [42]. The administration of Nigella sativa extract had a positive effect on bone healing by increasing cell migration, differentiation processes, extracellular matrix formation, and extracellular matrix organization [43]. Kara et al.’s study reported that TQ induced rapid formation of the new bone and increased the number of osteoblasts and the number of osteoclasts in a rat model. The systemic administration of TQ has been demonstrated to be effective in accelerating the formation of new bone in the rapid maxillary expansion (RME) method [13] and healing of the defects created in the skull of osteoporosis rats [41]. TQ induces bone repair by accelerating the differentiation of osteoblasts and activating BMP-2 (bone morphogenetic factor-2) [43]. Our study also confirmed that the local application of TQ in the scaffold can have positive effects on bone formation and its possible mechanism can be through the activation of growth factors such as BMP-2.

TQ has antimicrobial and anti-inflammatory effects [44]. Inflammation has a dual effect on the bone formation process. On the one hand, it leads to osteolysis and damage to bone regeneration, and on the other hand, it has pro-osteogenic effects [45]. Considering that in this study no inflammation was observed in any group and the presence of inflammatory cells such as lymphocytes was very limited, the anti-inflammatory effect of TQ cannot be attributed as its possible mechanism in increasing bone formation. It has also been shown that TQ improves bone formation by increasing angiogenesis and accelerating the expression of vascular endothelial growth factor [29]. This could be another potential reason for the increase in bone formation in our study. The systemic application of TQ showed higher capillary density on bone healing in rat tibia, indicating that TQ increased angiogenesis and thus accelerated defect healing [11]. However, we did not evaluate capillary density in our study, further studies in this field are recommended.

Despite all the therapeutic uses, the systematic use of TQ also has problems. Among other things, the bioavailability of this substance is low due to its lipophilicity. Increasing the dosage is also associated with side effects such as liver toxicity [46]. The side effects of its systematic use have led to its topical use in this study, and as a result, it reduces the dosage and its toxic effects and increases the bioavailability in the defect region.

Alginate is a hydrogel that is used as a drug delivery vehicle. Alginate is non-toxic and has good biocompatibility [47]. It has been reported that alginate can increase bone differentiation in MSCs in vitro [48]. However, in the present study, alginate did not have any effect on increasing bone formation.

Our study had some limitations. Since bone mineralization is one of the most important issues in bone regeneration, the lack of evaluation of calcium content, alkaline phosphatase activity, or gene expression level involved in mineralization such as osteocalcin can be the main limitations. Also, checking the level of angiogenesis with stereological methods, the use of micro-CT scanning to check the hard tissue formation, and studying the ossification signaling pathway, such as measuring BMP levels, are other limitations of this project.

## Conclusion

According to the results obtained from this study, it seems that TQ-loaded alginate has positive effects on bone healing. Alginate scaffold graft containing TQ was significantly associated with an increase in newly formed bone area, the number of osteocytes and osteoblasts, and a decrease in the amount of connective tissue in the injury area. It seems that the local application of TQ can accelerate bone formation as well as its systematic or in vitro applications; however, it needs further study to confirm and find the possible mechanism.

## Acknowledgment

The authors thank the vice-chancellery of Shiraz University of Medical Sciences, for supporting the research (Grant#25594). This is the manuscript-relevant thesis of Dr. Mohamad Mehdi Farahmand. We are also grateful to the Department of Oral & Maxillofacial Medicine of Shiraz University of Medical Sciences, Faculty of Dentistry, who helped us in the process of conducting the study.

## Conflict of Interest

The authors declare no conflict of interest.
